# Genomic alterations in a cohort of pediatric acute myeloid leukemia patients at two cancer centers in Colombia

**DOI:** 10.1007/s12185-022-03475-w

**Published:** 2022-10-24

**Authors:** Luz K. Yunis, Adriana Linares-Ballesteros, Gisela Barros, Johnny Garcia, Nelson Aponte, Laura Niño, Gloria Uribe, Edna Quintero, Jaime Perez, Leila Martinez, Juan J. Yunis

**Affiliations:** 1 Grupo de Patología Molecular, Universidad Nacional de Colombia, Bogotá D.C., Colombia; 2 Servicios Médicos Yunis Turbay Y Cía S.A.S., Instituto de Genética, Calle 86B # 49D-28, Of 305, Bogotá D.C., Colombia; 3 Unidad de Oncología/Hematología Pediátrica, HOMI Fundación Hospital Pediátrico La Misericordia, Bogotá, Colombia; 4 Grupo de Oncohematología Pediátrica, Universidad Nacional de Colombia- HOMI Fundación Hospital Pediátrico La Misericordia, Bogotá D.C., Colombia; 5 Unidad de Patología, HOMI Fundación Hospital Pediátrico La Misericordia, Bogotá D.C., Colombia; 6 Unidad de Hemato-Oncología, Clínica Infantil Colsubsidio, Bogotá D.C., Colombia; 7 Departamento de Patología, Facultad de Medicina E Instituto de Genética, Universidad Nacional de Colombia, Bogotá D.C., Colombia

**Keywords:** Pediatric, Acute myeloid leukemia, Genomics, Colombia, Latin America

## Abstract

Few studies identifying genomic aspects in pediatric acute myeloid leukemia patients in Latin American countries have been reported. The aim of this study was to identify genomic alterations, clinical characteristics and outcomes in a cohort of pediatric AML patients. This descriptive observational cohort study included patients with confirmed de novo acute myeloid leukemia up to 18 years of age. Cytogenetics and conventional FISH analysis, next-generation sequencing and PCR testing were performed. The correlation of genomic data with treatment response and outcomes were analyzed. Of the 51 patients analyzed, 67.4% had a cytogenetic abnormality and 74.5% had a genetic variant. *FLT3* variants (ITD or TKD D835) were found in 27.4%, followed by *NRAS* (21.6%), *KRAS* (13.7%) and *WT1* and *KIT* (11.8%). Patients were stratified by risk (66.6% high-risk) after the end of induction. FLT3-ITD was associated with relapse (OR 11.25; CI 1.89–66.72, *p* 0.006) and *NRAS* with death during induction (OR 16.71; CI 1.51–184.59, *p* 0.022). Our study highlights the importance of rapid incorporation of genetic testing in pediatric AML in Colombia, as it directly affects treatment decisions and outcomes. Incorporation of targeted therapies with conventional chemotherapy is an increasingly urgent need in pediatric patients.

**Supplementary Information:**

The online version contains supplementary material available at 10.1007/s12185-022-03475-w.

## Introduction

Acute myeloid leukemia (AML) is a heterogeneous neoplasm representing the second most common type of acute leukemia in pediatric age, with less favorable outcomes than acute lymphoblastic leukemia, reaching overall survival rates that do not exceed 70% in developed countries and relapse rates that vary between 25 and 50% [1, 2], these outcomes are less favorable in developing countries.

Genetic alterations and clinical characteristics in pediatric AML have been studied previously, the majority have been carried out in European and North American population (which includes Hispanics living in the United States), but there is little information about the biological characterization and clinical outcomes in Colombian pediatric patients with de novo AML, even less on the molecular aspects [3]. This lack of knowledge directly affects risk stratification, treatment and survival; hence the importance of conducting this type of studies in our population. The aim of this study was to describe and correlate the genetic alterations, clinical characteristics and outcomes in a cohort of pediatric patients with de novo AML in two pediatric cancer centers from Bogotá, D.C., Colombia.

## Methods

### Patients, diagnosis, treatment and risk stratification

Descriptive observational cohort study, fifty-one patients between 1 month and 18 years of age with a confirmed diagnosis of de novo acute myeloid leukemia (non-promyelocitic) were included by convenience, with prior informed consent between March 2015, and June 2021, from two main pediatric cancer centers of Colombia, HOMI Fundación Hospital Pediátrico La Misericordia and Clínica Infantil Colsubsidio, Bogotá, D.C., Colombia. Patients with Down syndrome or secondary AML were not included. This protocol was approved by the institutional ethics committee of the Universidad Nacional de Colombia, HOMI Fundación Hospital Pediátrico La Misericordia and Clínica Infantil Colsubsidio. Diagnosis was made on bone marrow aspirate sample, including morphology FAB classification and immunophenotyping by multiparametric flow cytometry (MFC) analysis using EuroFlow Panel. Institutional protocol is based on the national clinical practice guideline [4], all patients received two induction cycles of “7 × 3” with cytarabine 100 mg/m^2^/d on days 1–7 and daunorubicin 60 mg/m^2^/d on days 1, 3 and 5. Additionally, all patients received triple intrathecal chemotherapy as prophylaxis of CNS involvement on days 1 and 7. Risk stratification was based on cytogenetic and molecular criteria, along with treatment response (supplemental material).

Post-induction treatment (consolidation) consisted in chemotherapy with 2–3 cycles of intermediate dose of cytarabine 3 gr/m^2^/day during 3 days plus triple intrathecal chemotherapy in standard-risk patients. For patients within intermediate-risk group, with matched related donor received hematopoietic stem cell transplantation (HSCT) after one or two consolidation cycles and patients without a matched donor received chemotherapy with 2–3 consolidation cycles. Finally, the high-risk group received HSCT with the best available donor after one or two consolidation cycles.

### Outcomes and definitions

Response to induction treatment was defined as Complete Remission (CR) with less than 5% of blast by morphology after second induction cycle with complete hematological recovery (≥ 1000/µL leukocytes, ≥ 500/µL neutrophil granulocytes and ≥ 50,000/µL platelets), without blasts in peripheral blood and without extramedullary compromise; CR with incomplete hematologic recovery (iCR) as less than 5% blasts by morphology after the second induction cycle without hematological recovery as described above; Induction Failure (IF) as more that 5% of blasts by morphology in bone marrow after second induction cycle; Resistant Disease as failure to achieve complete remission after first line therapy which includes induction and consolidation (at least two cycles); Treatment Related Mortality was defined as death occurring during treatment and after achieving complete remission, and Transplant Related Mortality as death occurring during transplant process and not related to relapse [5, 6]. Relapse was defined as reappearance of blasts post-CR in peripheral blood, bone marrow or extra bone marrow locations without any other attributable cause. Death during induction was defined as patients dying during induction period or before hematological recovery after end of induction. Measurable residual disease (MRD) was evaluated by flow cytometry with 0.1% as cut-off level after day 21 of second induction cycle. Toxicity was defined according to Common Terminology Criteria for Adverse Events (CTCAE) [7] (supplemental material). Only toxicities grades 3 and 4 were described. Relapse-Free Survival (RFS) was calculated as the time from the first remission to relapse and Overall Survival (OS) was defined as the time from diagnosis to death or last contact alive [8]. For survival analysis, a two-year probability for relapse free survival (RFS) was calculated.

### DNA and RNA extraction

DNA was extracted from 200 µL of bone marrow using the QIAamp DNA Blood Mini Kit (Qiagen, Hilden, Germany) following the manufacturer's specifications. The DNA purity quantification and verification were performed using a NanoDrop™ 2000 spectrophotometer (Thermo Fisher Scientific, Waltham, Massachusetts, United States). The DNA obtained was stored at − 20 °C until use. RNA was isolated from bone marrow samples in EDTA using the Quick-RNA MiniPrep Plus kit (ZYMO RESEARCH, Irvine, CA, USA) following the manufacturer's recommendations. The extracted RNA was converted into cDNA using the High-Capacity cDNA Reverse Transcription kit (Applied Biosystems, San Francisco, CA, USA) following the manufacturer’s recommendations. The DNA purity quantification and verification were assessed using a NanoDrop™ 2000 (Thermo Fisher Scientific, Waltham, Massachusetts, United States). The RNA was stored at -80 °C, and the cDNA was stored at − 20 °C until use.

### Cytogenetics and FISH

Cell culture was performed to obtain metaphases for the chromosomal study with G and Q bands according to standardized protocols [9]. Chromosome visualization was performed using GenASIs (Applied Spectral Imaging, Carlsbad, CA, USA). At least 25 metaphases per sample were analyzed, and the nomenclature was described according to the recommendations of the International System for Human Cytogenomic Nomenclature (ISCN) 2020. FISH was performed to detect the following recurrent rearrangements: *t*(8;21) (ETO-AML1 [RUNX1-RUNX1T1] Translocation, Dual Fusion Probe, Cytocell, Cambridge, UK) and Inv(16) (CBFB/MYH11 Translocation, Dual Fusion Probe, Cytocell). At least 100 nuclei per study were analyzed, and the interpretation was performed by two independent observers using GenASIs. In some cases, an MLL [KMT2A] Breakapart Probe, Cytocell) was used to confirm cytogenetic findings. Cytogenetic nomenclature was described according to the ISCN 2020 recommendations.

### AML Gene panel and rearrangements by NGS

We analyzed 30 genes (*ABL1, ASXL1, BRAF, CALR, CBL, CEBPA, CSF3R, DNMT3A, ETV6, EZH2, FLT3, HRAS, IDH1, IDH2, JAK2, KIT, KRAS, MPL, NPM1, NRAS, PTPN11, RUNX1, SETBP1, SF3B1, SRSF2, TET2, TP53, U2AF1, WT1, ZRSR2*) using the Myeloid Plus kit by SOPHIA Genetics (Sophia Genetics SA, Saint Sulpice, Switzerland) panel by next generation sequencing (NGS) according to manufacturer’s recommendations. In addition, 119 fusions were studied with RNA Myeloid Plus Solution. Sequencing was carried out in an Illumina MiSeq (Illumina, San Diego, CA, USA) and sequencing analysis data with Sophia DDM^®^ software 5.2.7.1 (Sophia Genetics SA, Saint Sulpice, Switzerland)*. FLT3, NPM1* and *CEBPA* genes were also analyzed by rapid PCR testing as described previously [10–13]. Gene variants found by rapid testing in these three genes were confirmed by NGS. Subsequently, each variant was functionally annotated and categorized according to their pathogenicity, following the recommendations international consensus of the Association of Molecular Pathology, American Society of Clinical Oncology and the College of American Pathologists “Standards and Guidelines for the Interpretation and Reporting of Sequence Variants in Cancer” (2017) [14].

### Statistical analysis

Statistical analysis of quantitative variables is reported as means or medians with dispersion measures given in standard deviation and ranges, according to the nature and distribution of the variables, according to the Shapiro–Wilks normality test to establish the use of parametric tests or non-parametric. Qualitative variables were analyzed with Pearson’s Chi square test and Fisher’s exact test. Statistical analysis was performed using the Statistical Package for the Social Sciences (SPSS) for Windows, version 25.0. A value of *p* < 0.05 was considered significant. Statistical analyses of quantitative variables were carried out with measures of central tendency and dispersion, means and standard deviations, or medians and ranges, according to their distribution after analysis with normality tests (Kolmogorov–Smirnov or Shapiro–Wilk) to establish the behavior of the data as parametric or non-parametric. Medians were compared using the Mann–Whitney *U* test for independent non-parametric samples. For bias control, all diagnosed children entered the study, thus minimizing selection bias. Validated techniques were used, as well as positive and negative controls with sample processing. Information was double-checked when entered into the database for quality control.

## Results

### Clinical characteristics

Fifty-one patients were included. The median age was 10 years (range 0.15–18 years), with an M:F ratio of 1.42:1. Median leukocyte count at diagnosis was 25,580 (1190–1,896,000) and CNS involvement was present in 16 cases (31.37%). Patients were stratified as high risk 34 (66.6%) after the end of induction. During treatment, 96% of patients had at least one toxicity event, the most frequent were mucositis, colitis and transaminitis. Twenty-two out of 33 (66%) patients who received HSCT had a related toxicity event (Table 1).

**Table 1:** Demographic and biological characteristics

	*n*	%
Gender		
Female	21	41.1
Male	30	58.8

	%	IQ Range
Blasts	58.3	(24–82,8)

	*n*	%
Median age at diagnosis		
Female	21	41.2
Male	30	58.8
Total	51	100
WBC at diagnosis (mm^3^)		
< 20 × 10^9^/L	24	47
20 a 100 × 10^9^/L	15	29.4
> 100 × 10^9^/L	12	23.5
Cytogenetics		
t(8;21)	7	15.2
Inv16	5	10.8
*KMT2A* rearrangements	9	19.5
Complex karyotype	2	4.3
Other	10	21.7
Molecular		
FLT3	14	27.5
ITD	9	17.6
TKD	5	9.8
*NRAS*	11	21.6
Codon 12	5	9.8
Codon 13	2	3.9
Codon 61	4	7.8
*KRAS*	7	13.7
*WT1*	6	11.8
*KIT*	6	11.8
Exon 8	4	7.8
Exon 17	2	3.9
*CEBPA*	5	9.8
*U2AF1*	3	5.9
*NPM1*	2	3.9
*IDH1*	2	3.9
*PTPN11*	2	3.9
*ASXL1*	2	3.9
*ETV6*	2	3.9
*RUNX1*	1	2
*EZH2*	1	2
*CBL*	1	2
Treatment related toxicity		
Induction	46	92
Consolidation	36	81.8
HSCT	33	64.7
Autologous	9	17.6
Umbilical cord blood	14	27.5
Haploidentical	10	19.6
Treatment related toxicity	49	96
Mucositis	25	49
Colitis	24	47
Transaminitis	23	45
Cardiotoxicity	7	13.7
Aspergilosis	2	3.9
HSCT related	21	41.1
EICH	9	17.6
Treatment response		
MRD after First cycle 7 × 3		
< 0.1%	13	25.5
0.1 -10%	20	39.2
> 10%	8	15.7
Not available	10	19.6
MRD after second cycle 7 × 3		
< 0.01%	28	54.9
≥ 0.01%	14	27.5
Not available	9	17.6
Risk classification^a^		
Low risk	4	7.8
Intermediate Risk	8	15.6
High risk	34	66.66
Not available	5	9.8
Events and outcomes		
Remission	18	35.2
Relapse	14	27.5
Failure at end of induction	7	13.7
Death during induction phase	3	5.9
Death during treatment	4	7.8
Death before treatment	1	1.9
Toxicity related death	4	7.8
HSCT related death	9	17.6

*IQ* Interquartile

^a^End of induction

### Cytogenetics and FISH analysis

Five patients had a non-informative karyotype due to absence of metaphases to be analyzed. In the remaining 46 patients, 31 patients (67.4%) had either structural or numeric alterations, 8 (26%) had two or more chromosomal abnormalities (range 2–5). Twenty-four patients (51%) presented gene fusions detected by conventional cytogenetics and FISH. Eleven patients (23%) presented other numerical or structural alterations.

In 15 patients, karyotype or FISH analysis did not show any alteration (32.6%). Among the 31 patients with a cytogenetic alteration, 9 patients had *KMT2A* (MLL) gene rearrangements (19.6%); 7 patients had an *t*(8;21) (*ETO-AML1* [*RUNX1-RUNX1T1*] translocation (15.2%); 5 patients (10.9%) had inv(16)(*CBFB-MYH11*), and 10 patients had other structural or numeric gene rearrangements (21.7%) (Table 1).

### AML gene panel by next generation sequencing gene panel

Genetic variants were found either alone or in combination with other genetic variants by NGS in 38/51 patients (74.5%). Genetic variants above variant allele frequency (VAF) 5% were considered clinically relevant. The most frequent pathogenic genetic variant found was in *FLT3*. Fourteen patients (25.5%) had either ITD (8/14), TKD D835 (5/14) or both ITD/TKD (1/14). The second most frequent genetic alteration was in *NRAS* (codon 12, 13, 59 or 61) in 11 patients (21.6%), followed by *KRAS* variants in 7 (13.7%), *WT1* and *KIT* genetic variants in 6 patients, respectively (11.8%), *CEBPA* (5, 9.8%), *U2AF1* (3, 5.9%), *NPM1, PTPN11, ASXL1, ETV6* and *IDH1* (2, 3.9%), *RUNX1, EZH2* and *CBL1* (1, 2% each) (Table 1). Regarding *WT1* gene mutations, 2 patients had nonsense variants, 2 frameshift mutations and 2 patients had double mutations. It is worth it to mention that no *DNMT3A* mutations were found in our cohort.

### Genomic alterations, co-mutation patterns and clinical outcomes analysis

Eleven out of 12 patients with Core Binding Factor (CBF) alterations had mutations involving signaling pathways (*RAS/KIT/FLT3/WT1*). All *KIT* mutations are related to CBF fusions (4 with *t*(8;21) and 2 with inv(16)). Two out of 4 patients with *t*(8;21) and *KIT* mutations died (50%) (death related to HSCT). Patients with Inv(16) and *KIT* mutations are alive post-HSCT. In addition, 80% of patients with Inv(16) (4/5) carried *FLT3* gene variants (3 with TKD and 1 ITD)(2 dead related to HSCT, 1 in relapse, 1 alive in remission) (Tables 1 and 2).

**Table 2:**
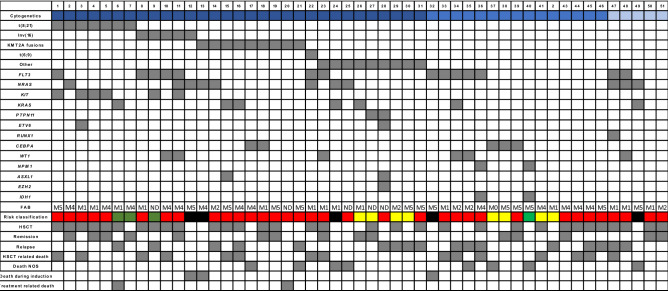
Distribution of genetic alterations, co-mutation patterns, treatment and outcomes in pediatric AML patient

*HSCT* Hematopoietic stem cell transplantation, *NOS* Non-other specified, *Dark blue* chromosomal abnormality, *blue* normal karyotype, *light blue* Non-informative Karyotype, *Red* High risk, *Yellow* Intermediate risk, *Green* Low risk, *Black* No risk stratification, *ND* not defined

**Fig. 1:**
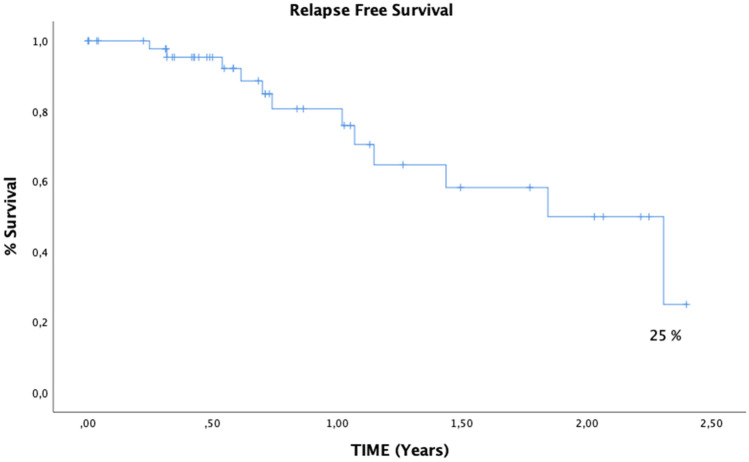
Kaplan–Meier curve for Relapse Free Survival

Twelve out of 14 patients (85.7%) with *FLT3* mutations were classified as high risk after the end of induction and received HSCT as part of their treatment. Four patients had a high allelic ratio (> 0.5) and 4 had a low allelic ratio. Only 3 out of 14 patients are alive and in remission (21.4%). We found significant association between FLT3-ITD with relapse 11.25 OR (CI 1.89–66.72, *p* 0.006) and lower blast clearance at day 21 after first induction cycle (3.69% median blast count in bone marrow range 0–55), while *FLT3* negative samples had a 1.35% median blast count in bone marrow (range 0–84). However, these differences did not reach a statistically significant difference. Six out of 9 *FLT3*-ITD patients died post-HSCT. All patients with high allelic ratio in FLT3-ITD that were classified as high risk after the end of induction died (2 due to relapse and 2 due to failed remission). Three out of four patients with low allelic ratio in FLT3-ITD died (2 relapse and 1 HSCT related death). No relationship was found with leukocyte counts or with a specific morphologic subtype. In 10 *FLT3* positive patients (71%) age was ≥ 10 years; there were no *FLT3* cases in children under 2 years of age, despite representing 25% of the cohort. All patients with *WT1* had *FLT3* mutations; four out of 6 patients died (66.6%). In addition, *NRAS* mutations were strongly associated with death during induction 16.71 OR (CI 1.51–184.59, *p* 0.022). No other significant associations were found in our cohort for other genetic variants (Table 3). Two-year probability for RFS was 25% in our cohort (Fig. 1).

**Table 3:** Frequent cytogenetic and molecular alterations and treatment related outcomes

	*t*(8;21)			Inv(16)			*KMT2A* fusions			FLT3-ITD			*NRAS*			*WT1*		
Events and outcomes	OR	CI 95%	*p*	OR	CI 95%	*p*	OR	CI 95%	*p*	OR	CI 95%	*p*	OR	CI 95%	*p*	OR	CI 95%	*p*
CNS	1.25	1.05–1.47	0.058	1.53	1.24–1.89	0.138	1.11	0.24–5.16	0.588	2.58	0.55–12.02	0.203	2.19	0.55–8.68	0.218	2.46	0.44–13.82	0.273
HSCT	2	0.21–18.92	0.476	1.36	1.11–1.61	0.332	2	0.21–18.92	0.476	2.42	0.61–22.49	0.39	2	0.21–18.92	0.476	1.61	0.16–15.63	0.571
Relapse	0.33	0.04–3.06	0.3	0.74	0.07–7.84	0.646	0.72	0.12–4.11	0.537	**11.25**	**1.89–66.72**	**0.006**	1.9	0.36–9.95	0.358	2.63	0.46–15.08	0.253
Induction failure	1.22	1.05–1.41	0.287	1.2	1.05–1.37	0.504	2.2	0.34–14.07	0.352	2.2	0.34–14.07	0.352	2.72	0.41–17.99	0.286	1.13	0.11–11.48	0.652
Death during induction	1.17	1.04–1.33	0.536	3.5	0.29–41.99	0.353	1.58	0.14–17.24	0.56	1.1	1.00–1.21	0.486	**16.71**	**1.51–184.59**	**0.022**	1.1	1.1–1.2	0.589
Death	0.52	0.10–2.60	0.343	0.15	0.18–7.57	0.632	1.65	0.36–7.50	0.393	6.68	0.76–59.04	0.061	2.41	0.56–10.44	0.197	1.6	0.26–9.64	0.476
Toxicity related to HSCT	0.94	0.14–6.25	0.65	1.58	0.18–2.12	0.19	0.67	0.09–4.79	0.528	1.25	1.19–7.92	0.599	0.94	0.14–6.25	0.65	0.25	0.34–1.79	0.176
GVHD	1.12	0.17–7.45	0.627	2.5	0.29–20.92	0.368	1.12	0.17–7.45	0.627	0.29	0.03–2.86	0.272	0.38	0.04–3.74	0.373	0.5	0.49–5.15	0.494
HSCT related death	1.42	0.21–9.58	0.533	3.14	0.37–26.62	0.295	3.5	0.56–22.02	0.188	0.38	0.39–3.65	0.365	1.5	1.14–1.95	0.122	0.63	0.06–6.48	0.582
MRD 21 > 0.1	3.27	0.35–30.46	0.271	1.44	0.13–15.33	0.623	1.19	0.20–7.16	0.611	1.19	0.20–7.16	0.611	1.19	0.20–7.16	0.611	2.6	0.27–24.94	0.368
MRD 21 > 1	1.97	0.33–11.63	0.374	2.28	0.28–24.08	0.445	1.97	0.33–11.63	0.374	1.97	0.33–11.63	0.374	1.97	0.33–11.63	0.374	4.21	0.45–39.85	0.191
MRD 21 > 10	0.64	0.07–6.25	0.584	1.27	1.07–1.51	0.404	0.64	0.07–6.25	0.584	**10**	**1.61–62**	**0.018**	1.86	0.29–12.01	0.416	6	0.93–38.52	0.077
MRD 42 > 0.1	0.77	0.13–4.55	0.571	0.64	0.06–6.79	0.593	0.77	0.12–4.55	0.571	1.25	0.25–6.22	0.543	3.33	0.63–17.65	0.153	1	0.16–6.25	0.689

*GVDH* Graft versus host disease, *HSTC* Hematopoietic stem cell transplantation, *MRD* Measurable residual disease

Statistical significant data is highlighted in bold

## Discussion

Despite growing data on pediatric AML and its genetic alterations, few studies have been carried out in the Latin American population outside of the US reflecting the clinical and molecular characteristics [15, 16]. Cytogenetic alterations in pediatric AML can be divided broadly into different specific groups: 25% CBF, 20% *KMT2A* rearrangements, and 20% normal karyotype. These alterations have a particular distribution pattern associated with age [17]. We found similar frequencies in the group of patients reported here with AML, CBF 26.1%, *KMT2A* 19.6% and normal karyotype 32.6%, respectively. In other countries of the region such as Peru, the frequency of cytogenetic alterations in a pediatric cohort from a reference hospital was recently described; 60.8% of patients had a chromosomal alteration, being *t*(8;21) the most frequent [15]. A report from Argentina showed similar results, 32% of normal karyotype, 17% of *t*(8;21), but discrepant for *KMT2A* 1.8% using only karyotype [16].

More than 90% of pediatric AML cases have at least one molecular alteration; the majority of cases with a normal karyotype [18]. The most frequent gene involved in pediatric AML is *FLT3* [10, 17–20]. A similar result was found in our cohort with 25.5% *FLT3* positive patients first by *FLT3* PCR and by NGS. However, in a report with more than 1000 AML patients, *NRAS* mutations were the most frequent mutations (nearly 30%) found in children with AML, followed by *FLT3* [18]. In another report, a low frequency of *FLT3* involvement in 27 Japanese patients was reported [21], however, the latter results should be taken carefully since NGS was used to characterize FLT3-ITD mutations, and unless the bioinformatic approach used is designed to detect large insertions, as the case of most FLT3-ITD mutations, it might be missed by NGS.

FLT3-ITD is known as an unfavorable prognostic marker [17, 19, 20, 22]. Our results corroborate this finding in Colombian patients, since 12 out of 14 patients (85.7%) were classified as high-risk patients at the end of induction and FLT3-ITD was found as a risk factor for relapse 11.25 OR (CI 1.89–66.72, *p* 0.006).

As mentioned previously, *NRAS* mutations was the most frequent mutation found among pediatric AML patients in one of the largest cohorts of AML pediatric patients studied thus far [[18]]. In our cohort, *NRAS* mutation was the second most frequent genetic alteration found (21.6%) and was strongly associated with death during induction 16.71 OR (CI 1.51–184.59, *p* 0.022), followed by *KRAS* (11.7%) and *WT1* (11.8%). Thus, *FLT3*, *RAS* and *WT1* were the most frequent mutations found in our cohort as has been reported earlier for a large cohort in the US [18]. Few studies have evaluated the clinical significance of *RAS* signaling pathway alterations in pediatric patients with AML. In the Japanese clinical trial AML-05, which includes more than 400 patients, *NRAS* was the most frequently involved, associated with a favorable prognosis, especially in the presence of *CBFB*-*MYH11* fusion [23]. In our cohort of 11 *NRAS* positive cases, 4 were also *FLT3* positive (3 ITD, one TKD), 2 had *CBFB*-*MYH11* fusion, and one *RUNX1-RUNX1T1* fusion. Currently, one *NRAS* positive patient with co-expression of *CBFB*-*MYH11* fusion and FLT3-TKD is alive and in remission.

Few studies have addressed the prognostic effect of *KIT* variants in pediatric patients [24]. The prognostic significance of *KIT* mutations in CBF is still controversial as a potential risk factor for prognosis [24]. In a systematic review, *KIT* mutations were related to relapse and poor relapse free survival, especially with FLT3*-*TKD D835 mutations. They were also seen at a higher frequency related to WBC increments, especially in patients with Inv(16) [25]. In a large study from patients between 16 and 64 years of age, a close association was found between adverse effects with *KIT* mutations and *RUNX1-RUNX1T1* but not with *CBFB-MYH11* [24]*.* In our cohort, all patients with *RUNX1-RUNX1T1* fusion and *KIT* mutations were classified as high risk after the end of induction (MRD > 1 after the first cycle of induction), 50% have died.

Co-occurrence of *WT1* with FLT3-ITD mutations are frequently associated with induction failure and dismal outcomes in children with AML (*p* < 0.0001) [2, 18]. In our cohort, we found that 6 patients carrying *WT1* mutations were also *FLT3* positive. However, this genomic relation did not show any statistically significant association with induction failure, likely due to the sample size of our study.

Despite treatment intensification with HSCT in patients with high-risk disease due to cytogenetic/molecular characteristics and poor response to induction therapy, outcomes continue to be unfavorable. In our cohort, 65% (33/51) of our patients had an indication for HSCT, currently 15 out of 33 (45%) are alive and in remission. However, in patients with FLT3-ITD mutations, this proportion was lower, 3 out of 14 patients are alive and in remission (21.4%). This highlights the importance of mutational genetic testing in addition to cytogenetic studies to characterize risk stratification and the incorporation of targeted therapy.

Recent studies evaluating different drug combinations and increasing doses have failed to improve outcomes and have increased toxicity in pediatric AML patients [26]. Several new therapeutic agents are currently used in Adult AML patients as targeted therapy [27]. Our results in pediatric AML patients show that molecular analysis, beyond conventional cytogenetic and FISH analysis, must be incorporated for a correct risk stratification and treatment. The incorporation of new agents for targeted therapy associated with conventional chemotherapy schemes is an increasingly urgent need, especially in developing countries like Colombia.

## Conclusions

To date, this is the first study in Colombian pediatric AML patients with a complete clinical and genomic characterization. Our study highlights the importance of a rapid and systematic incorporation of genetic analysis in pediatric AML in Colombia, as it directly affects treatment decisions and outcomes.

## Supplementary Information

Below is the link to the electronic supplementary material.

Supplementary file1 (DOCX 73 KB)
