# Supplementary material for: Genomic alterations in a cohort of pediatric acute myeloid leukemia patients at two cancer centers in Colombia
Source: Int J Hematol. 2022 Oct 24;117(2):269–77. doi: 10.1007/s12185-022-03475-w (PMC9889450; doi:10.1007/s12185-022-03475-w)
Supplement: Supplementary file 1 — Supplementary file1 (DOCX 73 KB) [file 12185_2022_3475_MOESM1_ESM.docx]

| **Risk group** | **Genetic Risk Criteria** | **Clinical Criteria** |
| --- | --- | --- |
| Standard Risk | - CBFβ - t(8;21)(q22;q22) - inv(16)(p13q22) or t(16;16)(p13;q22) - Biallelic CEBPα aberrations - NPM1 mutated – FLT3 wild type | - Genetic Standard Risk and   MRD <1% by Flow Cytometry at end of induction 1 or blast count <5% by morphology at end of induction 2 |
| Intermediate Risk | - Non-Standard Risk and Non-High Risk | - Non-Standard Risk and Non-High Risk |
| High Risk | - Complex karyotype (≥ 3 aberrations including at least one structural aberration) *excluding those with recurrent translocations* - 11q23/KMT2A rearrangements - inv(3)(q21q26)/t(3;3)(q21;q26) RPN1::MECOM - -7/del(7q) or -5/del(5q) - FLT3-ITD positive | - MRD >1% by Flow Cytometry at end of induction 1 or blast count >5% by morphology at end of induction 2 |

**Risk group definition by genetics and clinical criteria.**

**Toxicity definitions according to CTCAE version 5.**

| **CTCAE Term** | **Definition** | **Grade 1** | **Grade 2** | **Grade 3** | **Grade 4** |
| --- | --- | --- | --- | --- | --- |
| Oral mucositis | A disorder characterized by ulceration or inflammation of the oral mucosal. | Asymptomatic or mild symptoms; intervention not indicated | Moderate pain or ulcer that does not interfere with oral intake; modified diet indicated | Severe pain; interfering with oral intake | Life-threatening consequences; urgent intervention indicated |
| Colitis | A disorder characterized by inflammation of the colon. | Asymptomatic; clinical or diagnostic observations only; intervention not indicated | Abdominal pain; mucus or blood in stool | Severe abdominal pain; peritoneal signs | Life-threatening consequences; urgent intervention indicated |
| Transaminitis-  increased Alanine aminotransferase | A finding based on laboratory test results that indicate an increase in the level of alanine aminotransferase (ALT or SGPT) in the blood specimen. | >ULN - 3.0 x ULN if baseline was normal; 1.5 - 3.0 x baseline if baseline was abnormal | >3.0 - 5.0 x ULN if baseline was normal; >3.0 - 5.0 x baseline if baseline was abnormal | >5.0 - 20.0 x ULN if baseline was normal; >5.0 - 20.0 x baseline if baseline was abnormal | >20.0 x ULN if baseline was normal; >20.0 x baseline if baseline was abnormal |
| Transaminitis-  increased Aspartate aminotransferase | A finding based on laboratory test results that indicate an increase in the level of aspartate aminotransferase (AST or SGOT) in a blood specimen. | >ULN - 3.0 x ULN if baseline was normal; 1.5 - 3.0 x baseline if baseline was abnormal | >3.0 - 5.0 x ULN if baseline was normal; >3.0 - 5.0 x baseline if baseline was abnormal | >5.0 - 20.0 x ULN if baseline was normal; >5.0 - 20.0 x baseline if baseline was abnormal | >20.0 x ULN if baseline was normal; >20.0 x baseline if baseline was abnormal |
| Cardiotoxicity- Left ventricular systolic dysfunction | A disorder characterized by failure of the left ventricle to produce adequate output. | - | - | Symptomatic due to drop in ejection fraction responsive to intervention | Refractory or poorly controlled heart failure due to drop in ejection fraction; intervention such as ventricular assist device, intravenous vasopressor support, or heart transplant indicated |
| Cardiotoxicity- Heart failure | A disorder characterized by the inability of the heart to pump blood at an adequate volume to meet tissue metabolic requirements, or, the ability to do so only at an elevation in the filling pressure. | Asymptomatic with laboratory (e.g., BNP [B-Natriuretic Peptide ]) or cardiac imaging abnormalities | Symptoms with moderate activity or exertion | Symptoms at rest or with minimal activity or exertion; hospitalization; new onset of symptoms | Life-threatening consequences; urgent intervention indicated (e.g., continuous IV therapy or mechanical hemodynamic support) |
| Cardiotoxicity- Conduction disorder | A disorder characterized by pathological irregularities in the cardiac conduction system. | Mild symptoms; intervention not indicated | Non-urgent medical intervention indicated | Symptomatic, urgent intervention indicated | Life-threatening consequences |

**Treatment protocol for pediatric AML patients.**

**
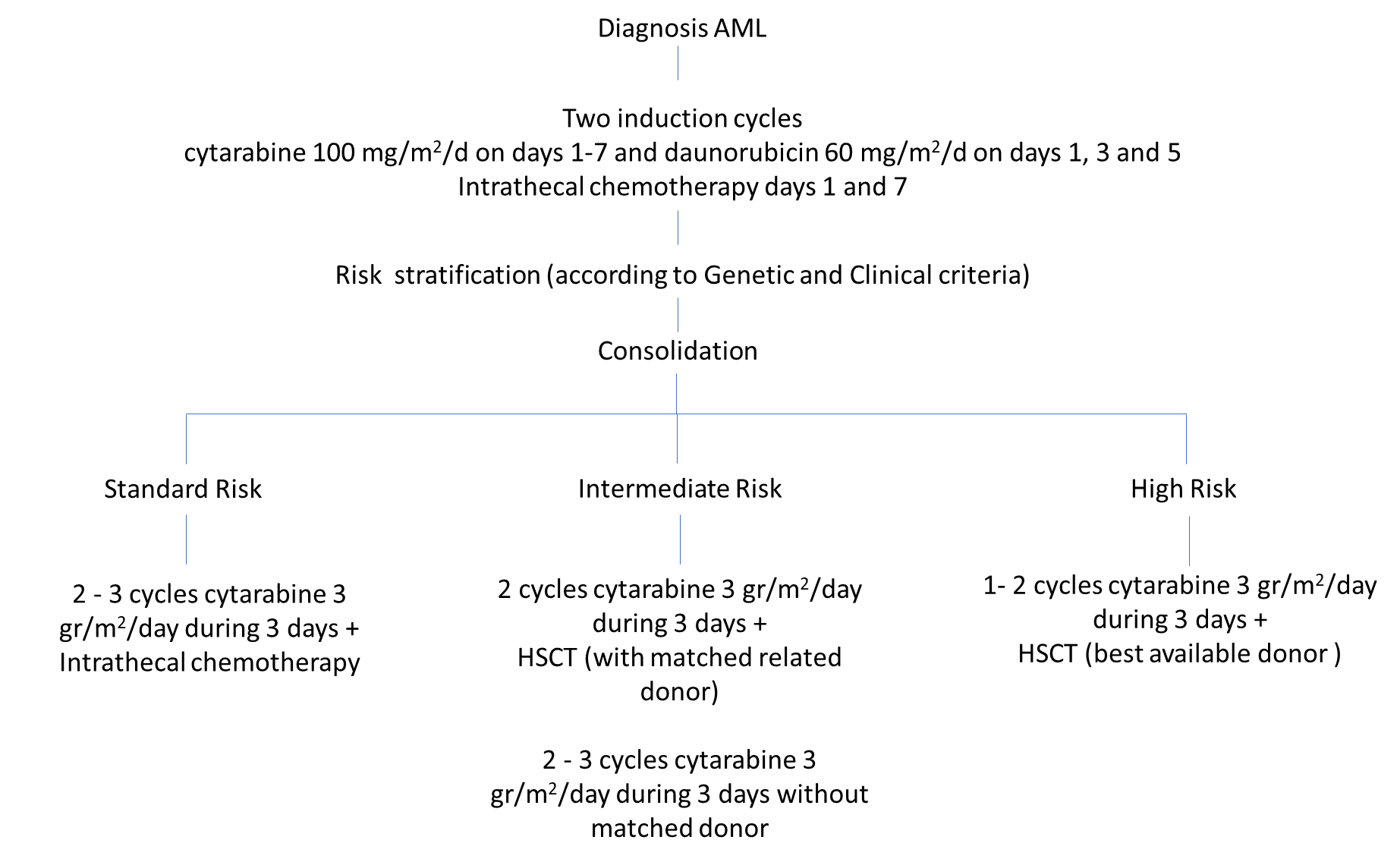
**
